# *In Vitro* Flow Rate Dependency of Delivered Dose and Fine Particle Dose of Salmeterol/Fluticasone Propionate Easyhaler and Seretide Diskus with Patient Flow Rates Collected in a Randomized Controlled Trial

**DOI:** 10.1089/jamp.2018.1463

**Published:** 2019-03-29

**Authors:** Rain Jõgi, Satu Lähelmä, Mikko Vahteristo, Anita Happonen, Jussi Haikarainen

**Affiliations:** ^1^ Lung Clinic, Tartu University Hospital, Tartu, Estonia.; ^2^ Orion Corporation, Orion Pharma, Kuopio, Finland.; ^3^ Orion Corporation, Orion Pharma, Espoo, Finland.

**Keywords:** delivered dose, fine particle dose, flow rate dependency, Salmeterol/Fluticasone Easyhaler

## Abstract

***Background:*** The Easyhaler^®^ device-metered dry powder inhaler containing Salmeterol and Fluticasone propionate (S/F) has been developed for the treatment of patients with asthma and chronic obstructive pulmonary disease (COPD). We report two studies which evaluated the *in vitro* flow rate dependence of delivered dose (DD) and fine particle dose (FPD) of S/F Easyhaler versus Seretide Diskus^®^.

***Methods:*** A randomized controlled trial (RCT) assessed inspiratory flow parameters of S/F Easyhaler and Seretide Diskus in subgroups of patients with asthma (children, adolescents and adults, and elderly) and in COPD patients. The 10th, 50th, and 90th percentile airflow rates were determined and utilized *in vitro*, to evaluate flow rate dependence of DD and FPD. Flow rate dependence was evaluated relative to the result obtained at the 50th percentile and any values deviating from 100% indicated flow rate dependence. The volumetric flow rate dependence (*Q*) index derived from FPD at 10th and 90th percentile airflows was also evaluated.

***Results:*** Overall, 227 patients were enrolled and randomized; 216 completed the RCT. In total, 55.5% of patients were female, and the mean age was 46.3 years. Clinically relevant airflow rates (46, 68, and 85 L/min for S/F Easyhaler and 44, 71, and 96 L/min for Seretide Diskus) were carried forward into the *in vitro* study, which demonstrated similar flow rate dependence of DD and FPD for S/F Easyhaler compared with Seretide Diskus; all values were within ±15% limits across the 10th, 50th, and 90th percentile airflow rates. *Q* index results suggested that both S/F Easyhaler and Seretide Diskus are medium airflow-dependent products.

***Conclusions:*** Similar *in vitro* flow rate dependence of DD and FPD was demonstrated for S/F Easyhaler compared with Seretide Diskus, across a range of clinically relevant airflow rates, collected from patients with asthma and COPD.

## Introduction

Inhalation is the preferred route of treatment administration in patients with chronic respiratory diseases such as asthma and chronic obstructive pulmonary disease (COPD). This is due to facilitation of direct exposure to the airways and efficacy observed at lower doses, compared with oral or systemic treatment options.^(1–3)^

Dry powder inhalers (DPIs) are among the most commonly used devices in patients with asthma and COPD.^(4)^ As discussed by Lavorini et al. several factors contribute to their effectiveness, including the patient's ability to generate sufficient inspiratory flow (required to deaggregate the powder particles to an optimal size to allow efficient lung deposition) and the resistance to airflow (which can impact directly on peak inspiratory flow [PIF]) within the device.^(4)^ Achieving dose accuracy, consistency across different inspiratory flow rates, and dose linearity at different dose strengths are also important requirements for DPIs.^(5,6)^

Concerning the flow rate resistance, the DPIs with a high built-in airflow resistance give the best lung penetration, because they enable a reduced velocity of the aerosol particles in the respiratory tract.^(7)^ Clinical experience shows that most patients can use a medium- to high-resistance DPI effectively, even during exacerbations.^(8,9)^

Ease of use of an inhaler is also critical for the effective management of these chronic respiratory diseases. Results from a recent systematic literature review of patients with asthma and COPD showed a high overall error frequency for both pressurized metered dose inhalers and DPIs (86.8% and 60.9%, respectively).^(10)^ Heterogeneity between studies (including the definition for critical error rates) meant that findings were limited. A need for a consensus on defining critical and noncritical errors have also been recently urged by Usmani et al.^(11)^ However, current asthma treatment guidelines also state that a high proportion of patients (up to 80%) have difficulty using their inhaler correctly.^(12)^ Together, these findings highlight current issues with ease of use in asthma and COPD and the need for adequate patient training before the use of inhalers.

Easyhaler^®^ (Orion Corporation, Orion Pharma, Espoo, Finland) is a device-metered DPI, which has been designed to be simple to use.^(3)^ Easyhaler has been reported to be easier to use compared with other inhalers in two studies of patient preference and two randomized, double-blind, double-dummy, parallel group studies of efficacy, involving children and adults with asthma or COPD^(13–17)^; a greater degree of user satisfaction was also reported for Easyhaler versus comparators in these studies.

In addition, the prominent Easyhaler mouthpiece (Supplementary Fig. S1; Supplementary Data are available online at www.liebertpub.com/jamp) meets the mouthpiece design objectives highlighted by Borgström et al.^(18)^ The patient should be able to easily obtain a good seal between the lips and the mouthpiece, and the mouthpiece should allow aerosol release deep enough into the mouth to avoid any interaction between the aerosol and the teeth or the tongue.

Current Easyhaler products approved for asthma and/or COPD include salbutamol, beclomethasone, budesonide, formoterol (monotherapies), and budesonide/formoterol (combination therapy). Salmeterol/Fluticasone propionate in combination is the most recent Easyhaler product developed for the treatment of asthma and COPD.

Current European guidelines on the required clinical documentation for orally inhaled products (OIPs) specify that characterization of flow rate dependence is required for DPIs within the range of clinically relevant pressure drops/flow rates. The Committee for Medicinal Products For Human Use guidelines for OIPs^(5)^ and pharmaceutical quality of inhalation and nasal products^(19)^ both describe the need to perform a product comparison using airflows that are characterized within the targeted patient population.

In line with the requirements detailed in these guidelines, the objectives of the current studies were to assess the inspiratory flow parameters of Salmeterol/Fluticasone propionate Easyhaler inhaler in patients with asthma and COPD, and evaluate the *in vitro* flow rate dependence of delivered dose (DD) and fine particle dose (FPD) of Salmeterol/Fluticasone propionate Easyhaler, compared with Seretide Diskus^®^ (GlaxoSmithKline, Brentford, United Kingdom).

## Methods

Two distinct, sequential studies were conducted: the first was an open randomized controlled trial (RCT) to evaluate the inspiratory flow parameters of placebo-filled Salmeterol/Fluticasone propionate Easyhaler and Seretide Diskus inhalers in patients with asthma and COPD. A subsequent *in vitro* study was carried out to evaluate the flow rate dependence of DD and FPD of Salmeterol/Fluticasone propionate Easyhaler and Seretide Diskus, using clinically relevant flow rates collected in the RCT.

### Randomized controlled trial (RCT)

#### Study design and treatments

This randomized, multicenter, crossover study (Orion study number: 3106002; ClinicalTrials.gov: NCT01424137) was performed at five centers (three in Estonia and two in Finland). Patients inhaled three times via three inhalers (Salmeterol/Fluticasone propionate Easyhaler A and B and Seretide Diskus in a randomized manner) to record their inspiratory flow parameters. Two Easyhaler inhalers were included because at the time of the RCT, both versions were still considered as options for the Salmeterol/Fluticasone propionate Easyhaler product. Easyhaler A has lower airflow resistance than Easyhaler B. Airflow resistance of the inhalers were 0.036, 0.044, and 0.027 kPa^0.5^ min/L for Easyhaler A, Easyhaler B, and Seretide Diskus, respectively.

At screening, spirometry was performed according to American Thoracic Society/European Respiratory Society guidelines (ATS/ERS; as reported by Miller et al.^(20)^); restricted activities specified by the ATS/ERS also applied.^(21)^ Native PIF rate, age, gender, height and weight, smoking history, medications (including the type of inhaler in current use and concomitant medications) were also recorded at screening.

Before measurement of inspiratory flow parameters, inhaler-specific training was given to all patients according to the instructions for use of each inhaler starting from residual volume. Patients subsequently practiced inhalation with placebo inhalers between one and three times per inhaler. After practicing, inspiratory flow parameters were measured using a SpiroMaster MX (Medikro, Kuopio, Finland) as described previously.^(22)^ Measurements were collected with patients in a standing position; however, they could be taken with patients in a seating position if required (e.g., for patients using wheelchairs). Three inspiratory flow curves per patient were recorded, and the best of the three measurements was analyzed.

All study procedures were performed at a single visit. The study protocol was reviewed and approved by the Ethics Committee for each of the centers and performed according to the principles outlined by the Declaration of Helsinki, Good Clinical Practice, and Good Manufacturing Practice guidelines.

#### Patients

Eligible patients had a documented diagnosis of asthma and/or COPD. Patients were excluded if they had any other severe chronic respiratory disease or acute respiratory infection, a medical condition, which could endanger them if they participated in the study (e.g., contraindication to spirometry), were lactose intolerant with subjective symptoms at lactose doses <0.5 g, or had severe milk allergy. Participants were also excluded if enrolled in a concurrent clinical study.

Patients were randomized to either the Diskus or Easyhaler, then randomly allocated to one of four treatment sequences designed by the study statistician (Supplementary Table S1). Randomization was performed at Orion Pharma by an independent randomization expert (Oracle Clinical Randomization). To ensure a random allocation of the inhaler sequences, all randomized patients received the inhaler sequence that corresponded to the next consecutive subject number, assigned at entry into the treatment period of the study.

All patients provided written informed consent before participating in the study. Parents provided written informed consent on behalf of any patients <18 years of age; in such cases, the investigator provided the patient and their parents with full, adequate verbal and written information regarding the objectives and procedures of the study, explained any possible risks and benefits involved before requesting the consent, and informed them of their rights to withdraw from the study at any time. Sufficient time was allowed for the patient and their parents to decide whether to participate in the study.

#### Endpoints and assessments

The primary endpoint was the PIF rate. Inspiratory volume, measured at the same time as the PIF rate, was the secondary endpoint. Both endpoints were evaluated in the following subgroups: patients with asthma 4–11 years (children); 12–64 years (adolescents and adults); ≥65 years (elderly); and patients with COPD of all ages.

### *In vitro* study

#### Endpoints and assessments

Flow rate dependence of DD and FPD using Easyhaler A, which was chosen as the final Salmeterol/Fluticasone propionate inhaler and Diskus inhalers, was assessed using the flow rate ranges achieved in the RCT. The minimum (10th percentile), median (50th percentile), and maximum (90th percentile) airflow rates were used for DD and FPD measurements. For both product strengths (50/250 and 50/500 μg/dose), a total of 72 inhalers from two batches of Salmeterol/Fluticasone propionate Easyhaler and a total of 84 inhalers from two batches of Diskus were used when comparing the effect of flow rate.

Flow rate dependence of the FPD was determined for the 50/250 μg/dose strength of Salmeterol/Fluticasone propionate Easyhaler and Seretide Diskus. To quantify the effect of inhalation flow on the FPD, Weers's and Clark's *Q* index was used as defined below:

\begin{align*}
Q { \rm { \;index } } = \bigg ( { \frac { { \rm { FPD \;90th \;percentile } } - { \rm { FPD \;10th \;percentile } } }  { { \rm { FPD \;higher } } } } \bigg ) \times 100
\end{align*}

The 50/250 μg/dose strength allows a direct comparison of our results with those from Weers and Clark who used the same dose for Seretide (Advair) Diskus.^(23)^ The formula used was modified for *in vitro* comparisons between inhalation products. Weers and Clark suggest using fixed pressure drops of 1 and 6 kPa for *Q* index determination,^(23)^ but instead, clinically relevant flow rates for both devices (as defined in the OIP and pharmaceutical quality of inhalation and nasal products guidelines^(5,19)^) were used.

FPD was also used in place of total lung dose. Using the *Q* index, the measured performance can be compared with the results found in the literature for example, Weers and Clark who provide this *Q* index information for also numerous other inhalation products. We consider this useful and believe that the use of *Q* index will become more common in the future for product evaluation.

### DD and FPD

DD and FPD were determined by using the sampling apparatus and procedures described in European Pharmacopoeia 8.0; for each flow rate evaluated, 4 L of air was drawn through the inhaler.^(24)^

Salmeterol and Fluticasone propionate collected in the sampling apparatus were analyzed by high-performance liquid chromatography method with UV detection at 280 nm. The chromatographic separations were carried out at 40°C on an Inertsil ODS-3 C18, 3 μm, 4.0 × 150 mm analytical column (GL Sciences) using 100 μL injection volume. The mobile phase, methanol:0.02 M phosphate buffer, pH 6.2 (25:75), was delivered at a flow rate 1 mL/mL. The samples were dissolved in water:acetonitrile 50:50 (v/v) and sample volume was 50 mL for DD samples and varied from 10 to 65 mL for FPD samples. The quantitation limit of the method for Salmeterol was 0.03 μg/mL and for Fluticasone Propionate, 0.1 μg/mL. The relative standard deviation of the peak areas varied not more than 2% during analysis.

For assessments of DD, a total of 36 devices were tested (three devices for each flow rate and strength/batch). Ten doses were measured for each inhaler, and the mean DDs were calculated.

FPD was defined as the amount of particles with an aerodynamic diameter ≤5 μm. FPD was determined using Next Generation Impactor (NGI, apparatus E).^(24)^ The cutoff points for impactor stages were calculated in relationship to the used flow rates according to European Pharmacopoeia 8.0,^(24)^ and FPD was derived from the data. For each FPD analysis, 10 doses were discharged into the NGI.

### Statistical analyses

The planned sample size in the RCT was 200–250 (≥50 patients per subgroup). As the primary objective of the study was to characterize the inspiratory flow parameters across the inhalers in the target patient population the sample size was not based on any power calculations and no formal statistical hypotheses were prespecified.

Patient demographics and characteristics were evaluated in all randomized patients; the primary and secondary endpoints were evaluated in the per protocol (PP) population. PIF rates and inspiratory volumes were presented as mean (standard deviation [SD]) with ranges). Results are only reported for Easyhaler A compared with Diskus, as this was the inhaler carried forward for the final Salmeterol/Fluticasone propionate Easyhaler product.

To provide flow rates for *in vitro* testing purposes, the achieved PIF rates through the Easyhaler and Diskus inhalers by the 10th, 50th, and 90th flow percentile airflows were weighted with the estimated proportion of asthmatic and COPD patients using long-acting β-agonists and inhaled corticosteroids combinations in Europe during 2012 (62% and 38%, respectively).^(25,26)^ This was required because the number of patients with COPD in the RCT was lower than in the overall population, to whom the product will be indicated.

Means and SDs were computed for DD and FPD collected at different *in vitro* flow rates. For assessments of flow rate dependence, the average DD and FPD for each product strength were divided by the value collected at the 50th percentile (median) airflow rate. A value differing from 100% at airflow extremes suggested that inhaler performance would be airflow dependent and the difference from 100% indicated the magnitude of airflow dependence. Differences between Salmeterol/Fluticasone propionate Easyhaler and Seretide Diskus were evaluated against ±15% limits.

Flow rate dependence for Salmeterol/Fluticasone propionate Easyhaler and Seretide Diskus was also calculated as a percentage, according to the *Q* index; the formula used can be modified for *in vitro* comparisons between inhalation products.^(23)^ We applied the 10th and 90th percentile airflow rate values, as well as FPD in place of total lung dose.

All data were analyzed descriptively; all analyses were performed using Statistical Analysis Software (SAS)^®^ for Windows version 9.3 (SAS Institute, Inc., Cary, NC).

## Results

### Randomized controlled trial

#### Patient demographics and characteristics

**FIG. 1.**
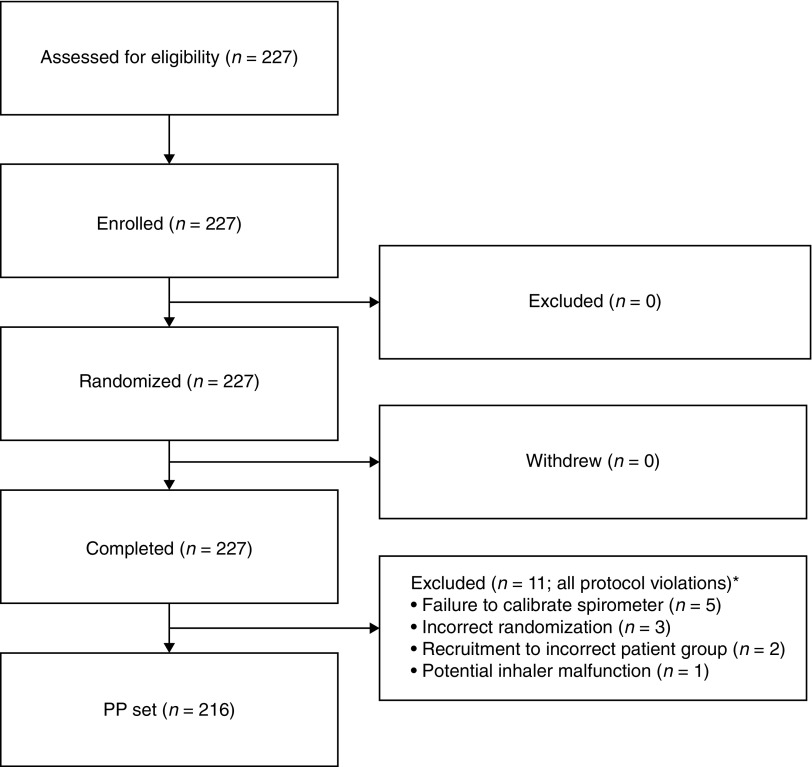
Patient disposition. *Six exclusions occurred in the subgroup of children with asthma; three patients were excluded from the subgroup of adolescents and adults with asthma; and one patient was excluded from each of the other two subgroups. PP, per protocol.

Overall, 227 patients were enrolled in the study and were randomized to treatment. A total of 216 patients completed the study according to the protocol and were included in the PP population (Fig. 1). Eleven patients were excluded from the PP population; the majority of exclusions were due to failure to calibrate the spirometer (*n* = 5) or incorrect randomization (*n* = 3).

Patient demographics and characteristics are shown in Table 1. The majority of patients randomized were female (*n* = 126; 55.5%), and the mean age (SD) was 46.3 (27.0) years; the mean age (SD) in the asthmatic children subgroup was 7.5 (2.5) years. Few patients (*n* = 6; 2.6%) had both asthma and COPD; 49 (21.6%) patients had allergic rhinitis in addition to asthma and/or COPD.

**Table 1.** Patient Demographics and Characteristics (All Randomized Patients)

	*Patients with asthma*			*Patients with COPD (*n = *53)*	*Total (*n = *227)*
	*Children (*n = *60)*	*Adolescents and adults (*n = *62)*	*Elderly (*n = *52)*		
Sex, *n* (%)					
Female	32 (53.3)	46 (74.2)	28 (53.9)	20 (37.7)	126 (55.5)
Age (years), mean (SD)	7.5 (2.5)	45.8 (15.9)	72.8 (5.3)	64.8 (7.4)	46.3 (27.0)
Range	4–11	13–64	65–88	50–82	4–88
Height (cm), mean (SD)	128.3 (16.2)	168.1 (10.0)	164.9 (9.4)	169.0 (8.8)	157.1 (20.8)
Weight (kg), mean (SD)	29.5 (11.5)	80.1 (19.7)	75.4 (15.1)	74.8 (17.3)	64.4 (26.5)
BMI (kg^2^/m^2^), mean (SD)	17.2 (2.9)	28.4 (6.8)	27.7 (5.0)	26.1 (5.5)	24.8 (7.0)
Race, *n* (%)					
Caucasian	58 (96.7)	62 (100)	52 (100)	53 (100)	225 (99.1)
Black	1 (1.7)	0	0	0	1 (0.4)
Hispanic	1 (1.7)	0	0	0	1 (0.4)
Previous/current use of nicotine products, *n* (%)					
Never used	60 (100)	41 (66.1)	35 (67.3)	1 (1.9)	137 (60.4)
Ex-user	0	11 (17.7)	13 (25.0)	20 (37.7)	44 (19.4)
Irregular user	0	1 (1.6)	1 (1.9)	0	2 (0.9)
Regular user	0	9 (14.5)	3 (5.8)	32 (60.4)	44 (19.4)
Respiratory, thoracic, and mediastinal disorders, *n* (%)					
Asthma	60 (100)	62 (100)	52 (100)	2 (3.8)	176 (77.5)
COPD	0	2 (3.2)	2 (3.9)	53 (100)	57 (25.1)
Allergic rhinitis	23 (38.3)	24 (38.7)	2 (3.9)	0	49 (21.6)
Pulmonary embolism	0	1 (1.6)	3 (5.8)	0	4 (1.8)
FEV_1_, L (SD)	1.6 (0.5)	2.7 (0.9)	1.7 (0.7)	1.4 (0.6)	1.9 (0.8)
% predicted FEV_1_ (SD)	101.2 (18.0)	82.1 (17.8)	69.2 (18.5)	47.2 (17.7)	76.0 (26.5)
Severity of airway obstruction, %					
FEV_1_ ≥80% of predicted	85.0	54.8	34.6	5.7	46.7
FEV_1_ ≥50% to <80% of predicted	15.0	41.9	50.0	34.0	34.8
FEV_1_ ≥30% to <50% of predicted	0	3.2	15.4	43.4	14.5
FEV_1_ <30% of predicted)	0	0	0	17.0	4.0
Previous/concomitant respiratory treatments, *n* (%)					
All treatments	56 (93.3)	60 (96.8)	51 (98.1)	50 (94.3)	217 (95.6)
Drugs for obstructive airway diseases	54 (90.0)	59 (95.2)	51 (98.1)	50 (94.3)	214 (94.3)
Nasal preparations	14 (23.3)	11 (17.7)	2 (3.9)	0	27 (11.9)
Antihistamines for systemic use	11 (18.3)	6 (9.7)	3 (5.8)	1 (1.9)	21 (9.3)
Cough and cold preparations	0	0	1 (1.9)	0	1 (0.4)

BMI, body mass index; COPD, chronic obstructive pulmonary disease; FEV_1_, forced expiratory volume in 1 second; SD, standard deviation.

The majority of patients (*n* = 217; 95.6%) were receiving therapy for asthma or COPD at the time of randomization; this was similar across all subgroups. Most patients in the subgroups of children (85.0%) as well as adolescents and adults with asthma (54.8%) had forced expiratory volume in 1 second (FEV_1_) ≥80% of the predicted value. In the group of elderly asthmatics, 50% of patients had FEV_1_ between 50% and 80% of the predicted value. COPD patients had the lowest FEV_1_ values; the majority had moderate (≥50% to <80% of predicted) or severe (≥30% to <50% of predicted) airway obstruction.

#### Primary endpoint: PIF

**FIG. 2.**
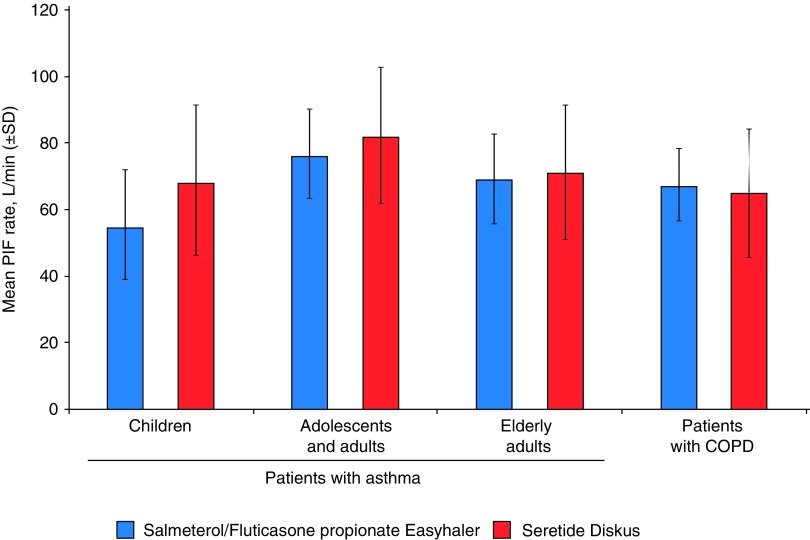
PIF rates (L/min) through Salmeterol/Fluticasone propionate Easyhaler and Seretide Diskus by patient subgroup (PP population; *n* = 216). COPD, chronic obstructive pulmonary disease; PIF, peak inspiratory flow; SD, standard deviation.

The mean PIF rates (L/min [SD]) for Salmeterol/Fluticasone propionate Easyhaler were lower compared with Seretide Diskus in children (54.4 [16.6] vs. 67.9 [22.6]) and adolescents and adults with asthma (75.8 [13.5] vs. 81.9 [20.5]). Mean PIF rates were similar for the tested products in elderly asthmatics (68.8 [13.5] vs. 70.9 [20.2]) and in patients with COPD (67.1 [11.0] vs. 65.1 [19.0], respectively) (Fig. 2).

**FIG. 3.**
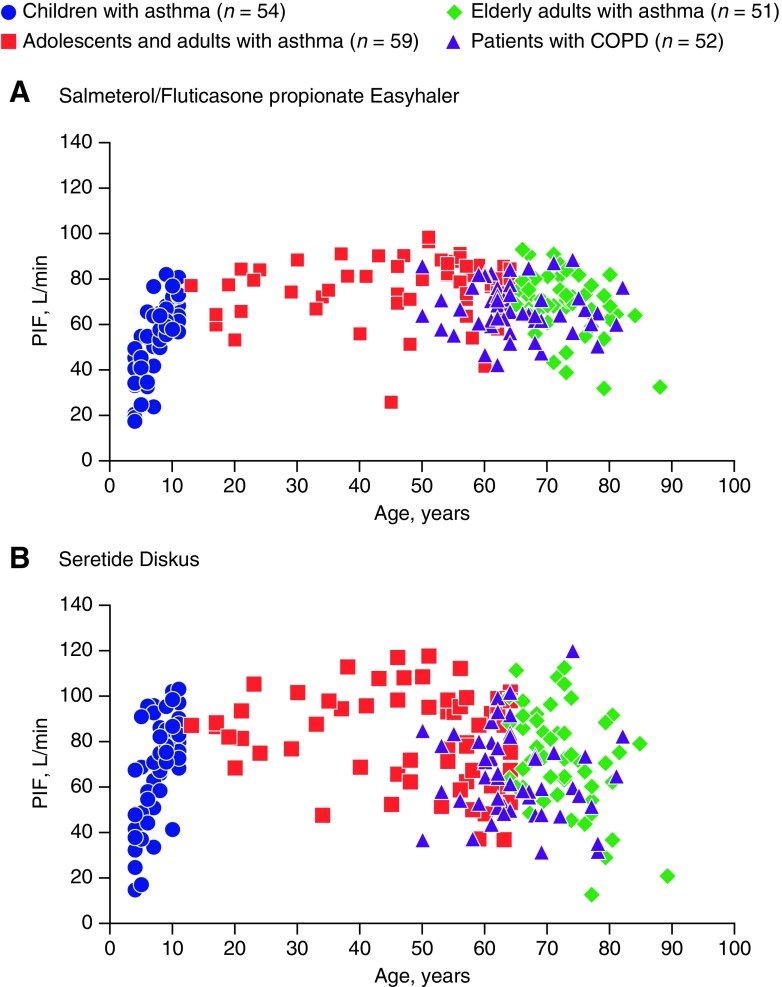
Individual PIF rates in each patient subgroup for Salmeterol/Fluticasone propionate Easyhaler **(A)** and Seretide Diskus **(B)** (PP population; *n* = 216).

For both inhalers, PIF increased with age in the subgroup of children with asthma; no clear trend was observed in the other patient subgroups (Fig. 3).

The weighted 10th, 50th, and 90th percentile airflow rates carried forward into the *in vitro* testing were 46, 68, and 85 L/min for Salmeterol/Fluticasone propionate Easyhaler and 44, 71, and 96 L/min for Seretide Diskus. The measured airflow rates correspond to pressure drops of 2.7, 6.0, and 9.4 kPa for Easyhaler and 1.4, 3.7, and 6.7 kPa for Diskus, when the widely referred formula by Clark and Hollingworth^(27)^ is used for measured airflow rates conversion, and reported airflow resistances are used.

\begin{align*}
\surd P \; = \;Q \; \times R ,
\end{align*}

where *P* is pressure drop, *Q* the airflow rate, and *R* the airflow resistance of the device.

#### Secondary endpoint: inspiratory volume

Mean inspiratory volumes (L [SD]) were similar for Salmeterol/Fluticasone propionate Easyhaler compared with Seretide Diskus in children with asthma (both 1.4 [0.6 vs. 0.5, respectively]), but slightly lower for Salmeterol/Fluticasone propionate Easyhaler in adolescents and adults with asthma (2.5 [0.7] vs. 2.7 [0.8]), elderly asthmatics (2.0 [0.7] vs. 2.3 [0.9]), and patients with COPD (2.0 [0.6] vs. 2.2 [0.7]).

### *In vitro* study

#### Flow rate dependency

The DD and FPD results for both strengths of Salmeterol/Fluticasone propionate Easyhaler and Seretide Diskus with different flow rates are shown in Tables 2 and 3, respectively.

**Table 2.** *In Vitro* Delivered Dose Across the Clinically Relevant Patient Flow Rates for Salmeterol/Fluticasone Propionate Easyhaler and Seretide Diskus

	*10th, 50th, and 90th percentile airflow rates, L/min*											
	*Easyhaler 50/250 μg/dose*			*Diskus 50/250 μg/dose*			*Easyhaler 50/500 μg/dose*			*Diskus 50/500 μg/dose*		
	*46*^a^*(*n = *60)*	*68*^b^*(*n = *60)*	*85*^c^*(*n = *60)*	*44*^a^*(*n = *60)*	*71*^b^*(*n = *60)*	*96*^c^*(*n = *60)*	*46*^a^*(*n = *60)*	*68*^b^*(*n = *60)*	*85*^c^*(*n = *60)*	*44*^a^*(*n = *60)*	*71*^b^*(*n = *60)*	*96*^c^*(*n = *60)*
Salmeterol												
Mean (μg/dose)	44	46	46	42	44	45	45	46	47	42	44	45
SD	1.8	2.2	3.4	2.0	2.3	1.8	2.3	2.4	2.2	3.2	2.6	2.1
RSD (%)	4.1	4.8	7.4	4.8	5.2	4.0	5.1	5.2	4.7	7.6	5.9	4.7
Fluticasone propionate												
Mean (μg/dose)	228	233	238	216	221	229	456	473	478	421	446	452
SD	14.9	11.8	16.2	10.2	10.9	8.4	25.7	29.1	22.8	32.4	26.2	18.8
RSD (%)	6.5	5.1	6.8	4.7	4.9	3.7	5.6	6.2	4.8	7.7	5.9	4.2

^a^ 10th percentile airflow rate.

^b^ 50th percentile airflow rate.

^c^ 90th percentile airflow rate.

RSD, relative standard deviation.

**Table 3.** *In vitro* Fine Particle Dose Across the Clinically Relevant Patient Flow Rates for Salmeterol/Fluticasone Propionate Easyhaler and Seretide Diskus

	*10th, 50th, and 90th percentile airflow rates, L/min*											
	*Easyhaler 50/250 μg/dose*			*Diskus 50/250 μg/dose*			*Easyhaler 50/500 μg/dose*			*Diskus 50/500 μg/dose*		
	*46*^a^*(*n = *6)*	*68*^b^*(*n = *6)*	*85*^c^*(*n = *6)*	*44*^a^*(*n = *6)*	*71*^b^*(*n = *6)*	*96*^c^*(*n = *6)*	*46*^a^*(*n = *6)*	*68*^b^*(*n = *6)*	*85*^c^*(*n = *6)*	*44*^a^*(*n = *12)*	*71*^b^*(*n = *12)*	*96*^c^*(*n = *6)*
Salmeterol												
Relative mean dose (%)^d^	82	100	108	86	100	104	77	100	111	87	100	105
SD (%)	3.4	4.7	3.3	3.2	7.1	10.9	3.2	5.1	5.0	4.2	4.4	3.4
RSD (%)	4.1	4.7	3.1	3.7	7.1	10.5	4.2	5.1	4.5	4.8	4.4	3.2
Fluticasone propionate												
Relative mean dose (%)^d^	88	100	106	86	100	105	82	100	108	88	100	104
SD (%)	2.8	3.9	2.7	2.2	5.2	7.6	2.6	4.9	5.1	4.5	4.4	3.7
RSD (%)	3.2	3.9	2.5	2.6	5.2	7.2	3.2	4.9	4.7	5.1	4.4	3.6

^a^ 10th percentile airflow rate.

^b^ 50th percentile airflow rate.

^c^ 90th percentile airflow rate.

^d^ The average FPD for each inhaler product/strength was divided by the corresponding value at median flow rate and multiplied by 100.

FPD, fine particle dose.

The DD for Salmeterol and Fluticasone propionate was similar between the products for both strengths, across the studied flow rate range.

The relative FPD values for Salmeterol and Fluticasone at 10th, 50th, and 90th percentile of clinically relevant patient flow rates are presented in Table 3. For Easyhaler, the relative mean (SD) FPD values for Salmeterol were 82% (3.4) and 77% (3.2) at the 10th percentile airflow rate for the 50/250 and 50/500 μg/dose strengths, respectively, lower than for Seretide Diskus (86% [3.2] and 87% [4.2]). Values for Salmeterol increased to 108% (3.3) and 111% (5.0) at the 90th percentile airflow rate for Easyhaler, and to 104% (10.9) and 105% (3.4) for Diskus.

The relative mean (SD) FPD values for Fluticasone propionate (Easyhaler) were 88% (2.8) and 82% (2.6) at the 10th airflow percentile using both product strengths, increasing to 106% (2.7) and 108% (5.1) at the 90th airflow percentile; values were similar to Diskus (105% [7.6] and 104% [3.7], respectively).

**FIG. 4.**
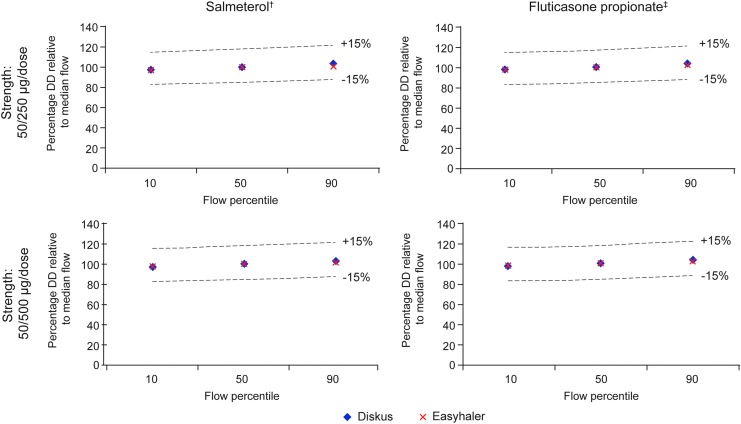
Relative *in vitro* dependency of Salmeterol and Fluticasone propionate DD at clinically relevant flow rates for Salmeterol/Fluticasone propionate Easyhaler and Seretide Diskus*. *10th, 50th, and 90th percentile airflow rates: 46, 68, and 85 L/min and 44, 71, and 96 L/min for Salmeterol/Fluticasone propionate Easyhaler and Seretide Diskus, respectively; ^†^Salmeterol strength: 50 μg/dose in both products; ^‡^Fluticasone propionate strength: 250 and 500 μg in the two respective products. DD, delivered dose.

**FIG. 5.**
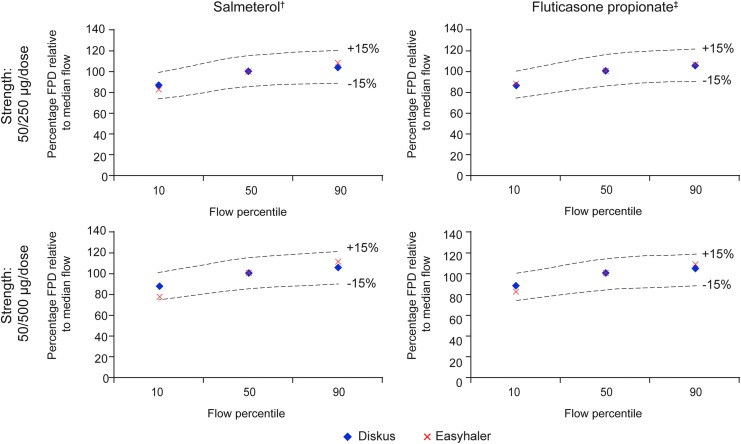
Relative *in vitro* flow rate dependency of Salmeterol and Fluticasone propionate FPD at clinically relevant flow rates for Salmeterol/Fluticasone propionate Easyhaler and Seretide Diskus. 10th, 50th, and 90th percentile airflow rates: 46, 68, and 85 L/min and 44, 71, and 96 L/min for Salmeterol/Fluticasone propionate Easyhaler and Seretide Diskus, respectively; ^†^Salmeterol strength: 50 μg/dose in both products; ^‡^Fluticasone propionate strength: 250 and 500 μg in the two respective products. FPD, fine particle dose.

Overall, flow rate dependence of DD and FPD of Salmeterol/Fluticasone propionate Easyhaler was similar to Seretide Diskus; all values were within ±15% limits across the 10th, 50th, and 90th percentile airflow rates (Figs. 4 and 5).

*Q* index values between 10th and 90th percentile airflow rates were +24.4% (Salmeterol, 90% confidence interval [CI] [22.1, 26.8]) and +17.1% (Fluticasone propionate [14.9, 19.3]) for Salmeterol/Fluticasone propionate Easyhaler 50/250 μg/dose. The corresponding results for Seretide Diskus 50/250 μg/dose were +16.3% [9.2, 23.5] and +17.7% [12.2, 23.3]. For the strength 50/500, the corresponding values were +30.2% (Salmeterol, [28.2, 32.2]) and +24.1% (Fluticasone propionate, [22.0, 26.2]) for Easyhaler and +16.9% [14.7, 19.1] and +15.7% [13.3, 18.1] for Diskus, respectively.

According to Weers and Clark, low, medium, and high flow rate dependence are defined by *Q* index values of 0%–15%, >15%–40%, and >40%, respectively.^(23)^ Results suggest that both Salmeterol/Fluticasone propionate Easyhaler and Seretide Diskus are medium airflow-dependent products.^(23)^

## Discussion

The main aim of these studies was to evaluate the *in vitro* flow rate dependence of DD and FPD for Salmeterol/Fluticasone propionate Easyhaler, compared with Seretide Diskus. Overall, similar flow rate dependence of DD and FPD was reported for the two products at both strengths, using the clinically relevant flow rates collected in the RCT. Therefore, lung deposition characteristics of the products are similar across a range of inspiratory capacities of the target patient population.

Flow rate independence of DD and dependence of FPD is a natural phenomenon. DD is independent when the same amount of drug is emitted from the device at all tested airflow rates. High FPD in turn also requires the power of airflow for deagglomeration of the drug particles from the carrier particles; the higher the flow rate, the higher the FPD for a positively airflow-dependent inhaler/formulation combination. This performance may be typical of nonair classifier-based inhalers, such as Easyhaler, Turbuhaler,^®^ and Diskus, which are positively airflow dependent.^(23)^

The data from our *in vitro* study suggest that DD and FPD were largely independent of airflow rate for both Salmeterol/Fluticasone Easyhaler strengths tested, with the FPD values relative to median flow between 77% and 111% from the 10th to 90th airflow percentiles; most values were of a similar magnitude to those for Diskus. Any small differences may be expected in the flow rate dependence for Easyhaler versus Diskus, due to differences in the design of the device and the lactose carrier, which may affect particle size distribution. Overall, all DD and FPD values relative to the median flow were well within ±15% limits, indicating similar flow rate dependence and no notable differences between the two products at each strength.

The *Q* index is a recently developed metric, which evaluates the degree of flow rate dependence as a percentage, based on the difference in total lung dose at flow rates corresponding to pressure drop values of 1 and 6 kPa.^(23)^

Our *Q* index analyses were carried out for the FPD values, and included the clinically relevant airflow rates derived from our *in vivo* study, as this would be in line with current European OIP guideline, which suggests 10th and 90th percentile airflow rates in addition to median as possible measurement points. This approach is justified as the realistic pressure drop values calculated for real patients using inhalers of different resistance differ from 1 and 6 kPa values utilized by Weers and Clark.

This approach also aligns the flow rate study to corresponding *in vitro* data. The pressure drop span for Easyhaler in the study was 6.7 kPa and for Diskus 5.3 kPa, where Weers and Clark used fixed 5 kPa pressure drop span (1 and 6 kPa). Our *Q* index results were comparable with the published results for the Diskus (+16.5% vs. +13.9% for Salmeterol and +18.2% vs. +21.7% for Fluticasone propionate, respectively),^(23)^ although applied pressure drops differed somewhat.

Similar to the findings of Weers and Clark, our data suggest that Seretide Diskus is a medium airflow-dependent product. Based on the results, we also report the same classification for Salmeterol/Fluticasone propionate Easyhaler, which was measured with wider pressure drop difference than Weers and Clark that is likely to increase the *Q* index figure somewhat compared to pressure difference from 1 to 6 kPa.

In the current RCT, differences in mean PIF rates included higher values for Seretide Diskus compared with Salmeterol/Fluticasone propionate Easyhaler in two subgroups of patients with asthma (children as well as adolescents and adults), but similar values in patients with COPD. Although these small numerical differences were observed, they were not evaluated further in this study.

Asthmatic children had the lowest PIF and inspiratory volumes of all patients assessed in our RCT. Two other studies reported by Azouz et al.,^(28,29)^ the other including Easyhaler and Diskus inhaler, also showed lower mean PIF rates and inspiratory volumes in children with asthma who received treatment with DPIs, compared with older patients. In our study, PIF rates increased with age in the subgroup of children with asthma (Fig. 3), whereas in the other subgroup, such improvement is not visible. Compared to our results, the lower measures of inspiratory flow reported earlier for both Easyhaler and Diskus inhalers might be at least partly due to lack of training before measurements.^(29)^ Indeed, enhanced training can result even up to 30% improvement in PIF rate.^(28)^

The study designs and the data collected met the current requirements for *in vitro* characterization and clinical documentation of OIP, for use in the treatment of patients with asthma and COPD.^(5)^ Also, a reasonable number of patients were enrolled (including those with mild-to-very severe airway obstruction), comparable with a recently published evaluation of flow rate dependence of DD and FPD,^(3)^ across all age groups for whom the product will be indicated. In addition, efficacy and safety of Salmeterol/Fluticasone propionate Easyhaler have been compared with Seretide Diskus in a pharmacokinetics study (NCT03060044) and found to be equivalent.^(30)^

Future work should also assess *in vitro* performance using realistic anatomic throat models and realistic patient airflow profiles.

## Conclusions

Similar *in vitro* flow rate dependence of DD and FPD was demonstrated for Salmeterol/Fluticasone propionate Easyhaler compared with Seretide Diskus, across a range of clinically relevant flow rates, collected from patients with asthma and COPD.

## Supplementary Material

Supplemental data

Supplemental data

## Notes

Reviewed by: Lavorini Federico, Gunther Hochhaus
