# Supplementary figures and images for: In Vitro Flow Rate Dependency of Delivered Dose and Fine Particle Dose of Salmeterol/Fluticasone Propionate Easyhaler and Seretide Diskus with Patient Flow Rates Collected in a Randomized Controlled Trial
Source: J Aerosol Med Pulm Drug Deliv. 2019 Mar 29;32(2):88–98. doi: 10.1089/jamp.2018.1463 (PMC6477585; doi:10.1089/jamp.2018.1463)

## Supplementary Data

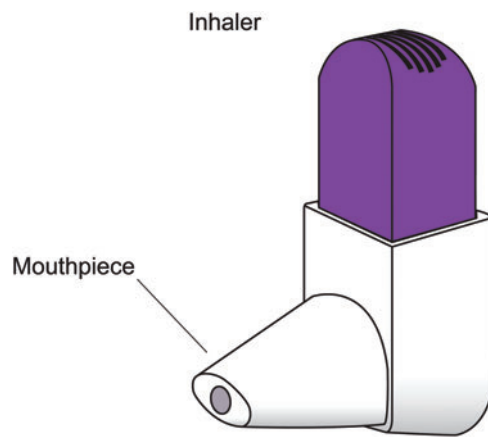

**SUPPLEMENTARY FIG. S1.** Easyhaler design.

Supplement: Supplemental data [file Supp_Fig1.pdf]
