# Supplementary material for: In Vitro Flow Rate Dependency of Delivered Dose and Fine Particle Dose of Salmeterol/Fluticasone Propionate Easyhaler and Seretide Diskus with Patient Flow Rates Collected in a Randomized Controlled Trial
Source: J Aerosol Med Pulm Drug Deliv. 2019 Mar 29;32(2):88–98. doi: 10.1089/jamp.2018.1463 (PMC6477585; doi:10.1089/jamp.2018.1463)
Supplement: Supplemental data [file Supp_Table1.pdf]

Supplementary Table S1. Treatment Sequences

Sequence	*Inhaler 1*	*Inhaler 2*	*Inhaler 3*
1	Diskus	Easyhaler A	Easyhaler B
2	Diskus	Easyhaler B	Easyhaler A
3	Easyhaler A	Easyhaler B	Diskus
4	Easyhaler B	Easyhaler A	Diskus
